# Distinguishing low frequency mutations from RT-PCR and sequence errors in viral deep sequencing data

**DOI:** 10.1186/s12864-015-1456-x

**Published:** 2015-03-24

**Authors:** Richard J Orton, Caroline F Wright, Marco J Morelli, David J King, David J Paton, Donald P King, Daniel T Haydon

**Affiliations:** Boyd Orr Centre for Population and Ecosystem Health, Institute of Biodiversity, Animal Health and Comparative Medicine, College of Medical, Veterinary and Life Sciences, University of Glasgow, Glasgow, G12 8QQ United Kingdom; Medical Research Council-University of Glasgow Centre for Virus Research, Institute of Infection, Inflammation and Immunity, College of Medical, Veterinary and Life Sciences, University of Glasgow, Glasgow, G12 8QQ United Kingdom; Pirbright Institute, Ash Road, Pirbright, GU24 0NF UK; Center for Genomic Science of IIT@SEMM, Istituto Italiano di Tecnologia at the IFOM-IEO Campus, Via Adamello 16, Milano, 20139 Italy

**Keywords:** Quasispecies, Viral population, Viral evolution, High-throughput sequencing, Next generation sequencing, Variant Calling, Sequencing error correction, RT-PCR Errors, Rare mutations

## Abstract

**Background:**

RNA viruses have high mutation rates and exist within their hosts as large, complex and heterogeneous populations, comprising a spectrum of related but non-identical genome sequences. Next generation sequencing is revolutionising the study of viral populations by enabling the ultra deep sequencing of their genomes, and the subsequent identification of the full spectrum of variants within the population. Identification of low frequency variants is important for our understanding of mutational dynamics, disease progression, immune pressure, and for the detection of drug resistant or pathogenic mutations. However, the current challenge is to accurately model the errors in the sequence data and distinguish real viral variants, particularly those that exist at low frequency, from errors introduced during sequencing and sample processing, which can both be substantial.

**Results:**

We have created a novel set of laboratory control samples that are derived from a plasmid containing a full-length viral genome with extremely limited diversity in the starting population. One sample was sequenced without PCR amplification whilst the other samples were subjected to increasing amounts of RT and PCR amplification prior to ultra-deep sequencing. This enabled the level of error introduced by the RT and PCR processes to be assessed and minimum frequency thresholds to be set for true viral variant identification. We developed a genome-scale computational model of the sample processing and NGS calling process to gain a detailed understanding of the errors at each step, which predicted that RT and PCR errors are more likely to occur at some genomic sites than others. The model can also be used to investigate whether the number of observed mutations at a given site of interest is greater than would be expected from processing errors alone in any NGS data set. After providing basic sample processing information and the site’s coverage and quality scores, the model utilises the fitted RT-PCR error distributions to simulate the number of mutations that would be observed from processing errors alone.

**Conclusions:**

These data sets and models provide an effective means of separating true viral mutations from those erroneously introduced during sample processing and sequencing.

**Electronic supplementary material:**

The online version of this article (doi:10.1186/s12864-015-1456-x) contains supplementary material, which is available to authorized users.

## Background

RNA viruses such as foot-and-mouth disease virus (FMDV) evolve rapidly due to their large population size, high replication rate and the poor proof-reading ability of their RNA-dependent RNA polymerase. Mutation rates of RNA viruses are cited to be between 10^−3^ and 10^−6^ mutations per nucleotide per transcription cycle [1-4], therefore mutations can potentially be introduced every time a viral genome is replicated. As a result, these viruses exist within their hosts as large, complex and heterogeneous populations, comprising a spectrum of related but non-identical genome sequences [5-8]. The capacity of RNA viruses to evolve rapidly is a key driver of viral virulence, vaccine resistance and host-jumping (the infection of a new species and adaptation to life within the new host). Therefore, an understanding of the mutational dynamics of RNA viruses is essential for our understanding of viral disease progression, transmission and the development of antiviral therapeutics [9].

Next generation sequencing (NGS) techniques provide the means for rapid and cost-effective dissection of viral evolutionary dynamics at an unprecedented level of detail [10-18]. The massively parallel and high-throughput nature of NGS platforms, combined with the relatively short genome of an RNA virus, enables the analysis of viral samples (which can contain billions of virions) to a very high depth. Such ultra-deep coverage of the genome enables the diversity of the whole viral population to be examined and subsequently compared between samples to investigate evolutionary events such as selection and bottlenecks. Furthermore, high depth enables the identification of important variants present within the viral population at low frequencies, such as those that increase pathogenicity or convey drug resistance [19] which would pose a risk if they become dominant in later populations due to selection. However, a current problem with the application of NGS platforms to viral population analysis is that true low frequency viral variants cannot be effectively distinguished from variants caused by errors during sample preparation or sequencing.

**Figure 1:**
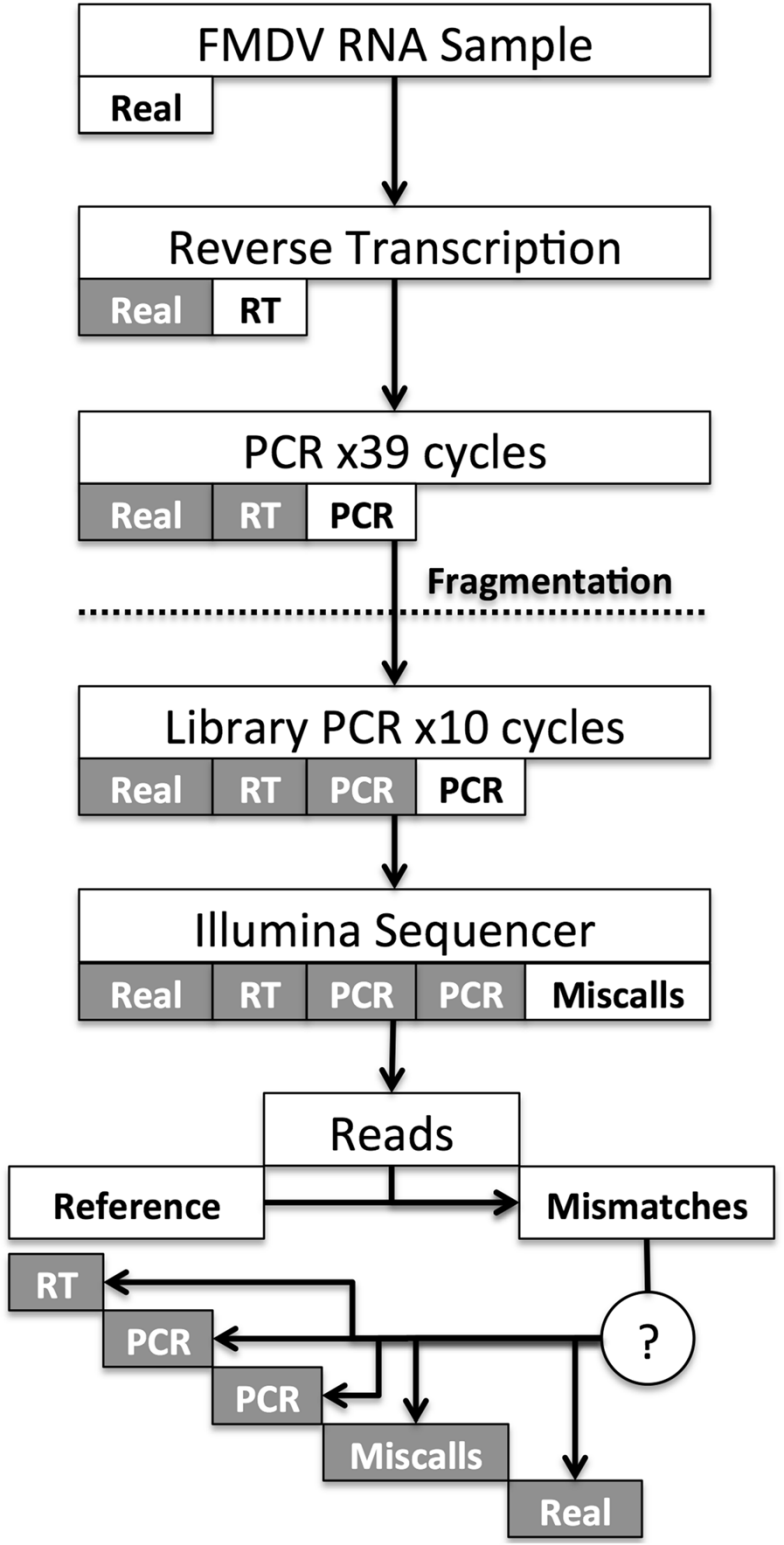
**RNA virus sequencing and error introduction.** The typical steps involved in processing an RNA virus sample for sequencing. At each step, errors are introduced in the form of artefactual mutations that will be present in the resultant NGS reads. Artefactual mismatches to the reference genome are practically indistinguishable from low-frequency real biological variation within the reads.

Viral samples typically have to undergo substantial sample processing before sequencing which introduces errors in the form of artefactual mutations into viral genomes (Figure 1). This problem is most evident for RNA viruses that must first be reverse transcribed (RT) to cDNA, which is then typically amplified by polymerase chain reaction (PCR) to produce sufficient quantities of DNA for sequencing on NGS platforms. The RT and PCR processes are error prone, with the error rate dependent on the fidelity of the enzymes used. RT is a non-expansive process, whilst PCR utilises multiple cycles to amplify the DNA in an exponential manner. In terms of error, the PCR process is cumulative, as any mutation introduced into a copied viral genome in one cycle will be passed on to all the progeny of that genome in later cycles in addition to new mutations being introduced. Therefore, there is an increase in the number of errors introduced into the viral sequences through the PCR process as the cycle number increases, elevating the error rate of the PCR process with each cycle.

Focussing on the Illumina platform, after RT and PCR, viral samples undergo fragmentation via sonication (or nebulization) to generate DNA fragments of various sizes. Typically 300-400 bp fragments are then selected by gel extraction. The process of sonication results in approximately half of the sample DNA being destroyed via evaporation, and only ~10% of the remaining DNA is located in the 300-400 bp range [20]. This results in a total sample loss of ~95% and therefore represents a sampling bottleneck of 5%. However, the latest Illumina Nextera DNA sample preparation kit simultaneously fragments and tags sample DNA with adapters in a single step (tagmentation) and results in significantly less sample loss [21] and hence requires substantially less starting DNA. Illumina library preparation involves the ligation of Illumina adaptors and unique sequencing tags onto the ends of DNA fragments followed by 10 cycles of PCR to amplify the library ready for sequencing on an Illumina platform.

Base calling on the Illumina platform is complicated by factors including similar emission spectra of the A/C and G/T fluorophores as well as phasing and pre-phasing issues [22]. In terms of error rate, the Illumina platform is considered to be better than its rivals [23-25] and every sequenced base in every read is assigned a quality score that is a measure of the uncertainty of the base call using the Phred scale. At one of the highest quality scores Q40, there is a 1 in 10,000 probability of a base being called incorrectly. Although this is a very low probability, the ultra-deep coverage of viral samples can result in a coverage of 10 K-100 K at each position in the genome. This implies that even if every base were of the highest quality, we would still expect errors to be introduced via base miscalls from the Illumina machine. Furthermore, it has been reported that Illumina machines have a sequence specific error profile whereby errors occur more frequently around certain motifs such as GGC and GGX [26-28]. Such systematic errors are poorly understood given that motifs such as GGC (the codon for glycine) occur very frequently in DNA, and yet it is only a small number of these motifs in a genome that suffer such errors, suggesting that there are other issues involved such as the DNA sequence further upstream of the motif.

The introduction of erroneous mutations during the sample preparation process and miscalls during base calling confounds the identification of true low frequency viral variants. Consequently, true low-frequency variants will be practically indistinguishable from process error, whilst high frequency viral variants will be easily identified, as they are observable at levels much higher than can be attributed to error. However, there have been few experimental analyses to determine thresholds above which a variant is highly likely to be real. Furthermore, there are very few computational methods available to distinguish true viral variants from erroneous mutations, with the majority of methods tailored to the 454 platform such as AmpliconNoise [29] and ReadClean454 [30]. Other tools aimed at detecting low frequency variants include Segminator II [31] which is specialised towards temporally sampled data, V-Phaser which utilises information on the co-location of variants on reads [32,33], Lo-Freq which utilises quality scores to model base miscalls [34], and an approach which incorporates re-sequencing with reads distribution and strand bias [35]. However, these tools do not consider the error effects of the RT or PCR processes; although Flux Simulator [36] does consider these processes it is specialised towards RNAseq datasets.

Here we focus on FMDV which has a genome of ~8.3 kb and is a non-enveloped, positive-sense, single-stranded RNA virus in the *Aphthovirus* genus of the family *Picornaviridae*. FMDV is the causative agent of the highly contagious and economically serious foot-and-mouth disease (FMD), a vesicular disease of cloven-hoofed animals, which can spread extremely rapidly and has the potential to cause enormous economic losses. In this study, we have created a set of novel control samples in the laboratory that are all derived from a DNA plasmid containing a full-length FMDV cDNA with extremely limited diversity in the starting population. One sample was ultra-deep sequenced on the Illumina platform without any RT-PCR sample processing, whilst the other samples were either PCR or RT-PCR amplified prior to sequencing. This enabled the level of error introduced by the RT and PCR processes to be individually assessed and also enabled minimum frequency thresholds to be set for true viral variant identification. We combined these experimental data sets with a genome wide computational model of the sample processing and NGS calling process to gain a detailed understanding of the error rates and thresholds at each step. Furthermore, we demonstrate how the model can also used to investigate a specific site of interest in any NGS data set, by utilising the site’s coverage, quality scores, and the fitted RT-PCR error distributions, one can investigate if the number of mutations observed are more than would be expected from processing errors alone. Combined, these data sets and the computational model provide an effective means of separating true viral mutations from those erroneously introduced during sample processing and sequencing.

## Methods

### Control sample and data preparation

**Figure 2:**
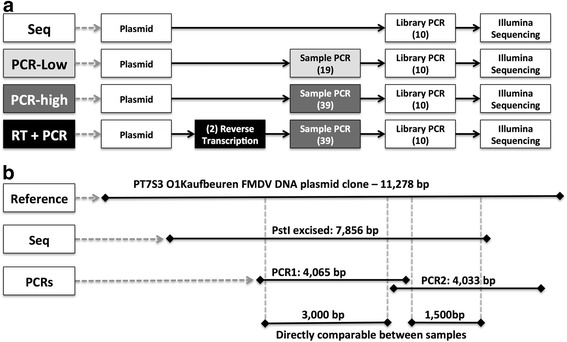
**Control sample processing and genome targets. (a)** is a schematic of the four different control samples and the processing that each one has undergone. The numbers in brackets represent the number of PCR cycles used during PCR steps, the (2) label on reverse transcription denotes that there was a preceding transcription step. **(b)** is a schematic of the DNA plasmid used, the genomic regions each of the control samples covers, and the regions that are directly comparable between all controls and used for later analyses.

Control sample preparation was performed in the high containment laboratory of the Pirbright Institute. The starting template used for all samples was an 11,278 bp pT7S3 plasmid containing a full-length FMDV B64 strain O1Kaufbeuren cDNA ([37], kindly provided by Veronica Fowler, Pirbright Institute). The DNA sequence of this plasmid clone had been determined previously by the Sanger method. This plasmid was clonally amplified in *E. coli* to create the necessary quantity of DNA for direct sequencing and as the starting template for all samples. Given the proof reading capabilities of bacterial DNA polymerases (estimated to have an error rate of between 10^−9^ and 10^−11^ errors per base pair copied [38]), the clonally amplified plasmid represents a relatively clonal starting population with a extremely limited diversity. Linearised pT7S3-O1K B64 plasmid (concentration of 10^9^ plasmid per μl) was generated using the restriction enzyme HpaI which in turn was used to generate four different control samples spanning three areas of error introduction (Figure 2):

1. Sequence: A 7,856 bp DNA fragment containing 80% of the FMDV genome was cut from the undiluted linearised pT7S3-O1K B64 plasmid using restriction enzyme digestion with PstI and then gel extracted. No RT or PCR was required to amplify this clonal sample.
2. PCR-low: Undiluted and linearised pT7S3-O1K B64 plasmid was subjected to two independent PCR amplifications, 19 cycles each, to generate 2 independent but overlapping PCR products - PCR1 and PCR2 - which are 4065 bp and 4033 bp in length, respectively. The DNA polymerase (DNApol) used in this PCR process was Platinum Taq High Fidelity (Invitrogen).
3. PCR-high: Linearised pT7S3-O1K B64 plasmid was diluted to 10^6^ plasmid DNA copies per μl (so as to result in the same amount of final DNA product as PCR-low), and then subjected to two independent PCR amplifications of 39 cycles each, to generate PCR products PCR1 and PCR2. PCR efficiency was measured with efficiency measured at 80% during the initial stages of PCR (see Additional file 1). The 10^6^ starting copy number and 39 cycles of PCR are the optimised values used to process FMDV samples for NGS sequencing determined from our previous studies [18], where further details of the laboratory protocol can be found.
4. RT-PCR: Linearised pT7S3-O1K B64 plasmid was diluted 1000 fold to 10^6^ plasmid DNA copies per μl, was first transcribed from DNA to RNA using the MEGAscript T7 kit (Ambion), and then reverse transcribed using Superscript III (Invitrogen) to generate cDNA followed by amplification via 39 cycles of PCR to generate PCR products PCR1 and PCR2, as above.

The Sequence control sample covers a slightly different portion of the plasmid genome than the remaining three PCR amplified samples due to the different location of the PstI restriction site to the previously optimised PCR primer sites (Figure 2); restriction site selection was influenced by biosecurity measures whereby no more than 80% of the FMDV genome could be transported out of the high security area for sequencing. To enable direct comparisons between all the samples, we focus our analyses on the portions of the FMDV genome that are shared between all of the samples (Figure 2). The ends of the DNA fragments are ignored due to large spikes in coverage at these positions, due to sonication bias.

Control samples were sequenced at the Polyomics Facility, University of Glasgow, on an Illumina Genome Analyzer IIx. Briefly, DNA was fragmented using sonication and the resultant fragment distribution assessed on an Agilent BioAnalyzer 2100 platform. After size selection of fragments of between 300 and 400 bp, a library of purified genomic DNAs was prepared by ligating adapters to the fragment ends to generate flow-cell-suitable templates. A unique 6-nt sequence tag for multiplexing was added to each control sample followed by PCR of 10 cycles using Phusion Oy (Finnzymes) DNApol. The amplified and tagged DNA samples were then pooled and attached to the flow cell by complementary surface-bound primers, isothermal bridging amplification formed DNA clusters for reversible terminator sequencing, yielding reads of 73 nucleotides.

Reads were processed using a previously developed pipeline [17,18]; however, other tools such as bwa [39] and bowtie [40] could be used as alternatives. Reads were first trimmed from 73 nt to 70 nt due to the very poor quality of the last 3 bases and then filtered, with reads discarded if they had an average probability of error per nt greater than 0.1%. The trimmed and filtered reads were then aligned to the plasmid reference genome with a simple, custom-made scoring algorithm, previously described in [17,18]. We further trimmed the first and last 6 nts of each aligned read, as they showed a higher number of mismatches to the reference sequence due to insertions or deletions close to the edges of the reads [17,18,20,41]. Information on the aligned reads is then piled-up to give the reference base, coverage and the number of As, Cs, Gs and Ts at each genome position along with an average probability of a sequencing error derived from the quality scores. The fastq files from all four samples have been uploaded to the EBI SRA repository (http://www.ebi.ac.uk/ena/) with the accession numbers ERR776658, ERR776657, ERR77656, and ERR776655 for the RT-PCR, PCR-High, PCR-Low and Sequence controls respectively.

### Computational model

The computational model operates at the population level. Instead of modelling 10^9^ or 10^6^ individual genomes (viral or plasmid) through the exponential PCR process, which would be computationally intensive, we view the population as an array or alignment of 10^6^ initial genomes and operate on the numbers of As, Cs, Gs, and Ts present at each genome position. This population view is based on the assumption that the copying of each individual base in each genome during PCR (or RT) is independent and is therefore a single binomial trial with a probability of success (a mutation) equal to the DNA polymerase error rate. Therefore, with 10^6^ genomes being duplicated in the first PCR cycle, each individual genome position is independently copied 10^6^ times with a mutation potentially introduced each time. An example of the alignment view and error accumulation through PCR cycles is presented in Additional file 1.

**Figure 3:**
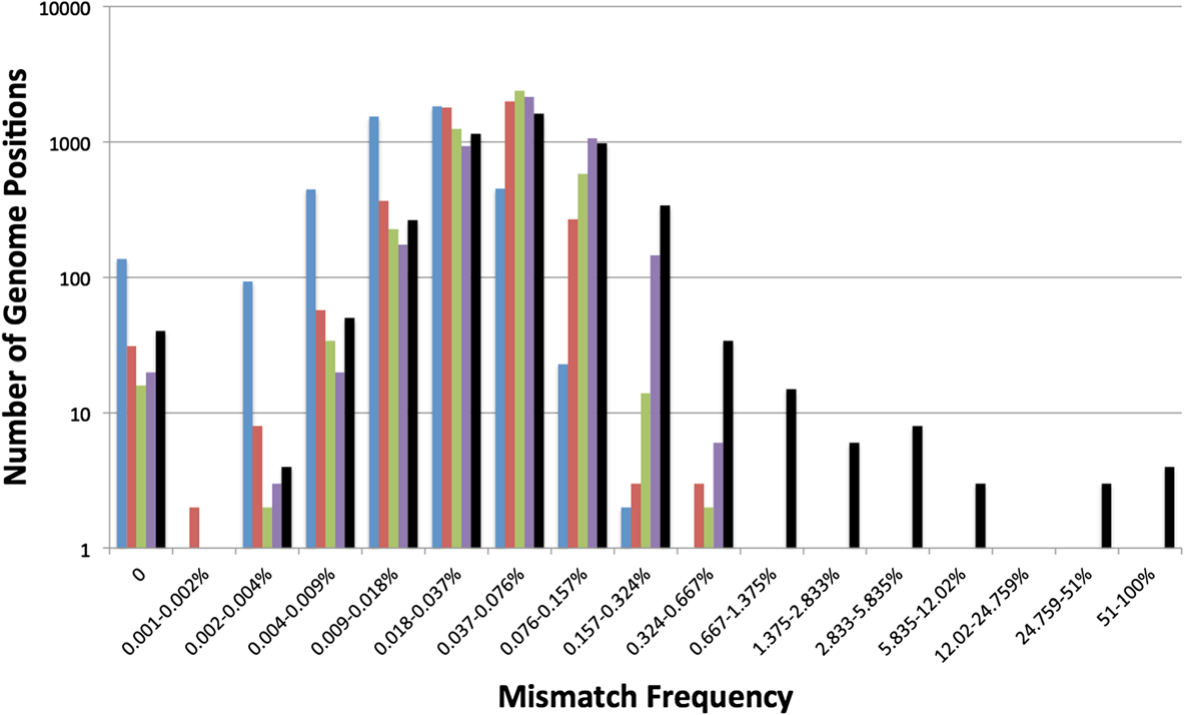
**Experimental data mutation spectra.** The mutation spectrum of each of the experimental samples: Seq (blue), PCR-Low (red), PCR-High (green) and RT-PCR (purple) along with a real FMDV sample (black) from an infected cow (A5-7DPFC-PB in [18]). Each genome position considered is placed into a discrete bin based on its mismatch (to the reference) frequency. The x-axis represents the mismatch frequency of the bin whilst the y-axis represents the number of genome positions that are in that bin. Both axes are presented on a log_10_ scale.

After simulation, the model gives a polymorphic/mismatch frequency (number of non-reference bases/total bases) for each genome position, which we present in the form of a mutation spectrum. The spectrum is generated by grouping genome positions into discrete bins based on their observed mismatch frequencies and then plotting the proportion of nucleotide sites in each mismatch bin (y-axis) against the mismatch frequency of the bin (x-axis; e.g. Figure 3). This spectrum provides a richer view of the diversity within a viral population, and enables easy comparison between populations. The mutation spectrum outputted from the model is then compared to the mutation spectrum from the corresponding experimental data set to give a sum of squares score (S) representing how well the model recreates the experimental data:

$$ S={\displaystyle {\sum}_1^b{\left({E}_i-{M}_i\right)}^2} $$

Where b is the number of polymorphic bins, and E_*i*_ and M_*i*_ are the number of nucleotide sites (represented as a proportion of all sites) in the *i*th polymorphic bin, for the experimental (E) and model (M) mutation spectrums, respectively. For model fitting and parameter estimation, we use a Sequential Monte Carlo (SMC) algorithm [42-45], a form of Approximate Bayesian Computation (ABC). We use the SMC algorithm proposed by [43], further details of which are presented in the Additional file 1, but briefly a parameter (or particle) θ is sampled from a prior distribution π(θ) and propagated through a sequence of intermediate distributions π(θ|d(x_0_, x^*^) ≤ ε_i_) until it represents a sample from the target distribution π(θ|d(x_0_, x^*^) ≤ ε_T_). d is the distance between the model simulated dataset (x^*^) and the laboratory data set (x_0_) calculated by the sum of squares score above. The tolerances ε_i_ are minimum distance scores and are chosen such that ε_i_ > … > ε_T_ ≥ 0, thus the distributions gradually evolve towards the target posterior. For the model fitting, we used uniform prior distributions and a dynamic tolerance schedule that progressively decreases the tolerance at each step. The SMC process essentially fits the model parameters to the given data set, and thus provides estimates of the error rates of the enzymes used during RT and PCR, as well as the underlying distributions involved.

The model is composed of a number of sample processing steps enabling various combinations to be selected for a simulation (the parameters at each step can be either manually set at the simulation start or dynamically fit by the model to the provided NGS data set):

1. Set Initial Diversity: The first step is to set the initial diversity of the viral population. This can be set to Clonal (no diversity), Fixed (all genome positions have the same initial mismatch frequency, or Sampled where the initial mismatch frequency of every genome position is drawn from a Beta distribution (α_S_, β_S_) where α_S_, β_S_ are the two shape parameters of a Beta distribution; α_S_, β_S_ can either be defined or added to the parameters to be estimated. For this study, we use the Clonal (no diversity) setting.
2. Determine RT enzyme error profile: Assuming the RT enzyme is more likely to make errors (mutations) at some genome positions than others (in a sequence specific manner) the RT enzyme error rate for each genome position is randomly selected from a Beta distribution (α_R_, β_R_). Alternatively, one can simply use a single RT enzyme error rate for all genome positions.
3. Transcription and Reverse Transcription: As our starting clonal sample is DNA, we first transcribe our DNA to RNA so that it can be reverse transcribed back to cDNA, enabling us to evaluate errors that impact upon the RNA template. We therefore represent this process as two distinct steps but where transcription uses the same parameters as reverse transcription, as we are unable to disentangle these two processes from each other using the data sets available. Transcription and reverse transcription are linear copying processes, with the original template removed after completion. Each genome position is considered in turn, where the population at each of the 4 bases (A, C, G, and T) at that position is also considered in turn. Error introduction is modelled by a binomial distribution (n_*ij*_, p_R*i*_), where p_R*i*_ is the RT enzyme error rate at genome position *i* and n_*ij*_ is the number of the *j*th base currently at genome position *i*. A random draw from this distribution gives the number of errors made, each error is randomly assigned to one of the other 3 bases at that genome position whose population increases by one, whilst that of the transcribed base decreases by one.
4. Determine DNApol error profile: Assuming the DNApol enzyme is more likely to make mutations at some positions than others, the DNApol error rate for each genome position is also randomly selected from a Beta distribution (α_D_, β_D_). Alternatively, one can simply set the DNApol error rate to be the same for all genome positions.
5. Sample preparation PCR: Each genome position is considered in turn, where the population at each of the 4 bases (A, C, G, and T) at that position is also considered in turn. First, if PCR efficiency is not 100% the number of bases to be copied is determined from a binomial distribution (n_*ij*_, p_C_), where p_C_ is the PCR efficiency for PCR cycle *c* and n_*ij*_ is the number of base *j* at genome position *i*; the population of base *j* is then increased by the number of bases to be copied. Second, the number of mutations that were made during copying is determined from another binomial distribution (n_*ij*_, p_M*i*_) where p_M*i*_ is the DNApol error rate at genome position *i* and n_*ij*_ is the number of the *j*th base copied at genome position *i* (determined from first step); Poisson and then normal approximations to the binomial are used when the population of n_*ij*_ is high (>100 and >10^9^, respectively). Third, the population of the copied base is decreased by the number of mutations to be made, and for each mutation the resulting mutated base is randomly selected to be one of the 3 other bases whose population increases by 1.
6. Sonication: The 5% sampling bottleneck caused by sonication is represented by a hyper-geometric distribution (which describes the probability of *k* successes in *n* draws). Here we use the Poisson approximation of the hyper-geometric for speed, and the corresponding normal approximation when the base population (*n*) is large. A Poisson distribution (λ_*ij*_) is used if the base population is <10^9^ where λ_*ij*_ is the mean and equal to [5% × population at genome position *i* base *j*]; for base populations >10^9^ we use the normal approximation of the hyper-geometric.
7. Illumina Library PCR: The 10 cycles of PCR performed during the Illumina library preparation proceeds in the same manner to the sample preparation PCR above, except that the DNApol error rate is fixed for each genome position to the published error rate of Phusion Oy (Finnzymes; 1 in 2,272,727).
8. Illumina Sampling: The reads obtained from an Illumina sequencing run represent a small sample of the amplified Illumina library. To represent this bottleneck, a sample of bases is randomly selected stochastically from the population present at each genome position, with the number of bases to be sampled given by the read coverage at that genome position from the corresponding experimental data set.
9. Illumina Error: Every base in every read is assigned a quality score (Q) by the Illumina machine, which can be converted into a probability of error (*p*) with the formula *p* = 10^-^^*Q*/10^. For each genome position, a mean probability of error can then be calculated from all the quality score *p* values aligned at that position. The number of Illumina errors at each position is then modelled by randomly drawing from a binomial distribution (n_*i*_, p_E*i*_) where p_E*i*_ and n_*i*_ are the average probability of a sequencing error and the coverage at genome position *i*, respectively.

## Results

### Data analysis

**Figure 4:**
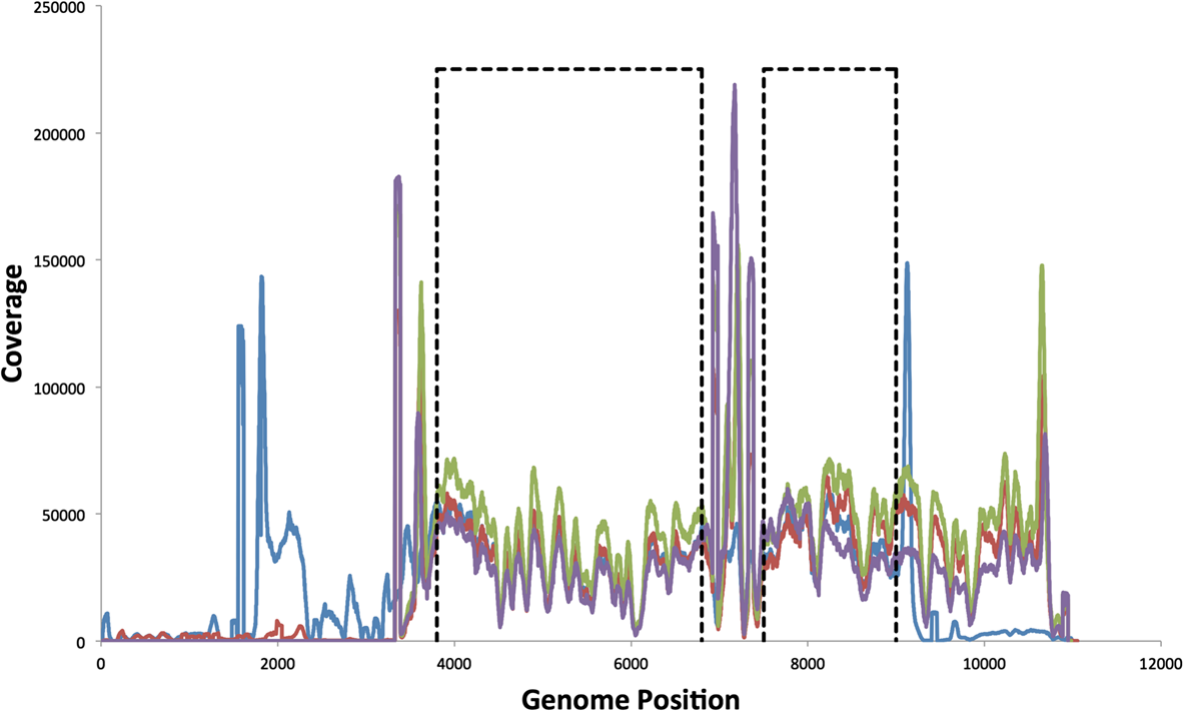
**Genome Coverage.** The read coverage for each of the samples across the 11,278 bp plasmid genome: Seq (blue), PCR-Low (red), PCR-High (green) and RT-PCR (purple). The peaks correspond to the ends of DNA fragments, which are over-represented in the data set, presumably due to sonication bias; as can be seen the Seq sample operates on a slightly different section of the genome (Figure 2) but with substantial overlap with the PCR amplified samples. The two regions of the genome that are used for direct comparison between all samples are highlighted with dashed black boxes, which avoid the regions with large and potentially biased coverage spikes at the ends of DNA fragments and primer regions. The Seq control (blue) suffers a drop in coverage at around position 2,500 due to a large poly C tract that is found in all FMDV genomes, and which is problematic for both PCR and sequencing.

After read filtering and alignment, coverage was high and similar in all four samples with an average coverage of 34,000 in the Seq control sample (Figure 4). Although coverage is variable across the genome, this variability is reproducible as all four samples display the same peaks and troughs in coverage at the same positions suggesting coverage is related to the underlying genome sequence. This variation correlates well with GC content (Additional file 1) and has previously been reported to influence coverage due to amplification bias during the Illumina library preparation PCR [46]. The very high peaks in coverage observed in Figure 4 occur at the end of the DNA fragments (both excised and PCR amplified) and are over-represented in the data set, presumably as a result of sonication bias at the end of DNA fragments.

Figure 3 displays the mutation spectra of the four experimental samples and represents a novel data set that measures the amount of error introduced during each stage of processing an RNA viral sample for sequencing, with the mutation spectrum shifting progressively towards higher error frequencies the more processing the viral sample undergoes. For the Seq control sample, the only source of error is from the Illumina machine and this error is still clearly observable despite read and alignment filtering; error from the Illumina library PCR step is predicted to be negligible due to the high fidelity of the DNApol used (see Additional file 1). The addition of PCR to the sample preparation shifts the mutation spectrum substantially to higher frequencies, and the addition of RT shifts the spectrum further still. The median mismatch frequencies of each spectra are presented in Table 1, along with the frequency at which 75%, 95% and 100% of genome positions are below. These frequencies can be used to set minimum thresholds above which one can be confident that observed mutations are real. For example, the maximum mismatch frequency observed in the full RT-PCR sample is 0.66%, whilst 95% of genome positions are below a frequency of 0.14%. The mutation spectrum from a real FMDV sample obtained from a foot lesion of an infected cow is also included in Figure 4, this sample underwent the same processing as the RT-PCR sample (minus the initial DNA to RNA transcription step). The spectrum of the real FMDV sample is similar to the clonal RT-PCR sample at low frequencies which represents the error introduced from sample processing and base calling, but it clearly diverges away from the clonal samples at a frequency of 0.5% bin onwards which therefore represent real biological variants in the viral population. There is undoubtedly real low frequency viral variation below 0.5% in the sample due to nature of viral replication, however this is practically indistinguishable from process error at this level.

**Table 1:** **Variant frequency thresholds**

	**25%**	**50%**	**75%**	**95%**	**100%**
**Seq**	0.0119%	0.0183%	0.0268%	0.0466%	0.276%
**PCR-Low**	0.0261%	0.0371%	0.0518%	0.0793%	0.4074%
**PCR-High**	0.0323%	0.0457%	0.0623%	0.1004%	0.3755%
**RT-PCR**	0.0368%	0.0526%	0.0793%	0.1403%	0.6571%
**Real**	0.0309%	0.0510%	0.0916%	0.2113%	100%

This table contains the 25^th^, median 50^th^, 75^th^, 95^th^ and the maximum 100^th^ percentiles of genome position mismatch frequencies in each of the four samples.

Table 1 shows that the maximum observed mismatch frequency in the RT-PCR sample was 0.66%; in the mutation spectrum this genome position is placed in the bin that has a midpoint of 0.5% (Figure 4). We can be highly confident that any mutation observed above this frequency is true, for samples processed with this protocol. However, there are likely to be a number of true viral variants present below this threshold. Figure 4 shows that the real FMDV sample begins to deviate from the RT-PCR sample at the 0.157% threshold, with more variants being observed in the real sample from this point on. A total of 226 variants are observed above this threshold in the RT-PCR control sample, whilst 516 variants are observed in the real FMDV sample, 290 of which are therefore likely to be true. A threshold of 0.66% would correctly identify all the errors (100% specificity), but would only identify 32 of the 290 likely variants in the real FMDV sample as true. Lowering the threshold by half to 0.3% results in a doubling of the number of likely true variants identified to 75 at a relatively small cost with specificity only dropping to 99.86%. Whilst at a threshold of 0.15%, specificity drops to 96% with 226 errors falsely identified as true, but all 290 are the likely true variants also identified. It is important to note that a threshold will be dictated by the actual sample processing used and must also be taken in context. The fidelity of the enzymes used and the number of the PCR cycles will highly influence threshold setting, whilst the coverage of a genome position and the quality of it’s aligned reads will influence a threshold in a site specific manner.

Next we characterised all the base substitutions that occurred in each of the clonal samples. In the Seq control sample, by far the most frequent base miscall was G to T (a G miscalled as a T) which represented approximately 23% of all Illumina base miscalls, followed by T to C, C to T, G to A and A to G which varied between approximately 12% and 15% (Additional file 1). Over 40% of all Illumina errors occurred at G positions, even though there were less G positions in the genome sequence considered than A or C positions; the remaining 60% of errors were distributed equally between the A, C and T positions. This suggests that the Illumina machine is more prone to error at G positions and could reflect the known issues with GGC and GGX motifs discussed previously. For the PCR samples, the most common base substitutions are those representing transitions between the purines (A to G and G to A) and pyrimidines (T to C and C to T), all of which are dominant within the dataset; although the A to G and T to C transitions are more abundant. Although substantially lower than these transitions, the G to T is by far the highest transversion, highlighting the contribution of the above Illumina error into the overall mutation spectrum. The RT sample shows the same characteristics as the PCR sample.

Overall, the mutation spectra suggest that Illumina error is clearly observable and should therefore be considered when examining viral populations at ultra-deep coverage. The main bulk of the error appears to come from the PCR process rather than the RT step, but RT does substantially affect the high frequency end of the spectrum. Although RT enzymes have higher error rates than their PCR counterparts, PCR utilises numerous amplification cycles and as errors are cumulative the amount of PCR error in the sample increases with each cycle.

### Computational model

**Figure 5:**
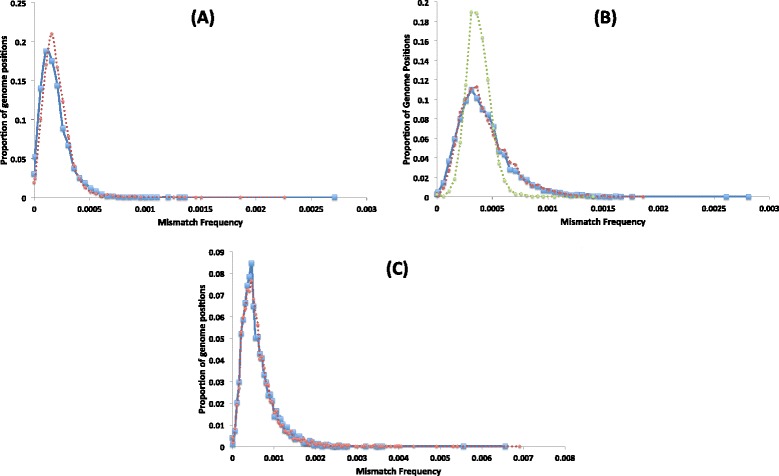
**Model simulated data mutation spectrums.** The mutation spectra of simulated data sets compared to its corresponding experimental data set: **(A)** Seq experimental (solid blue) and simulated (dashed red) and **(B)** PCR-High experimental (solid blue), simulated fixed DNApol error (dashed green), and simulated distributed DNApol error (dashed red); **(C)** RT-PCR experimental (solid blue) and simulated distributed RT error (dashed red).

We next developed a computational model of the sample preparation and sequencing process to parameterise each process and the errors that are introduced. In the model, the Seq control sample starts with an initial population containing no diversity, this is then sonicated (5% sampling), has library prep PCR amplification of 10 cycles, and is sampled at each position by the corresponding coverage in the real data set. Illumina base miscalls are represented via random draws from a binomial distribution based on the coverage and probability of sequencing error (derived from quality scores) at each genome position. This relatively simple approach to address Illumina error has been used successfully on FMDV [17,18] and other systems [47]. Figure 5A shows that there is ample agreement between the simulated and experimental data sets for the Seq control sample but the model slightly overestimates the amount of error introduced via Illumina miscalls. One possible explanation is that the probability of error associated with each quality score should in fact be lower (better quality) than the published value. This was also suggested in [9], who observed significantly lower mismatch rates than predicted from Illumina, quality scores when using overlapping paired-ends.

**Figure 6:**
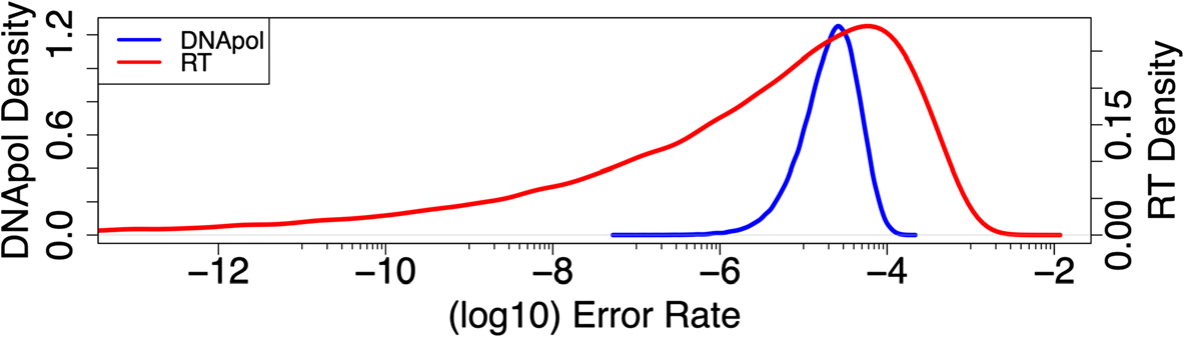
**Beta distributions representing enzyme error rates.** The model derived beta distributions representing the error rates for the DNA polymerase (blue line) and Reverse Transcriptase (red line) enzymes. 100,000 samples were randomly drawn from each Beta distribution. All error rates are logged (base 10) and the x-axis is truncated at −11 (the RT distribution has a long low density tail stretching to −30) to aid viewing and focus on the differences between the two distributions.

For the PCR-high model, there is poor agreement between the simulated and experimental data sets when one assumes that the DNApol error rate is the same for all genome positions (Figure 5B). When genome positions have the same mutation rate, there is not enough diversity in the predicted mutation spectrum. However, when one assumes that the DNApol enzyme is more likely to make mutations at some positions than others, through a Beta distribution, there is good agreement to the experimental data (Figure 5B). This could be due to the sequence composition of the surrounding bases influencing the likelihood of the DNApol making an error at certain positions. The model predicts that the median error rate of the DNApol used (Platinum Taq High Fidelity; Invitrogen) to be 2.20 × 10^−5^ substitutions per nt copied from the fitted Beta distribution (2.01, 76254), which is only slightly higher than the manufacturers published error rate of 1.8 × 10^−5^ (6 times the error rate of standard taq; Invitrogen). This Beta distribution results in variation between genome positions, with some positions slightly more or less prone to error during each PCR cycle than others (Figure 6), which will be magnified the more PCR cycles are used.

Similarly, for the RT-PCR model, there is again good agreement between the simulated and experimental data sets when one assumes the RT enzyme is more likely to make mutations at some positions than others (Figure 5C). The model predicts a highly skewed beta distribution (0.19, 3074 to represent the error rate of the RT enzyme used (Superscript III – Invitrogen), with a low median error rate of 5.31 × 10^−6^ but with a mean an order of magnitude higher at 6.13 × 10^−5^ substitutions per nt copied (Figure 6), which is within the range of published error rates for Superscript III (6.66 × 10^−5^ to 3.12 × 10^−5^; Invitrogen and [48]) which again varies depending on the assay used. The 95th percentile of this distribution has an error rate of 3.2 × 10^−4^ suggesting some genome positions are highly prone to error during the reverse transcriptase process. This could be due to sequence specific effects or perhaps the 2D/3D structure of the RNA influencing where the reverse transcriptase makes an error.

**Figure 7:**
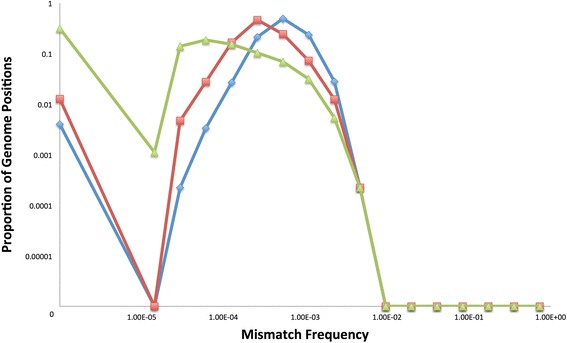
**Predicted mutation spectrums with higher fidelity polymerase and the true diversity of a real sample.** The predicted mutation spectrum of the full RT-PCR sample (blue), RT with no sample preparation PCR (red), and RT with no sample preparation PCR or Illumina error (green).

Through model fitting, we now have a good understanding of each of the sample processing stages along with their enzyme error rates and underlying distributions involved. As sequencing is always performed with DNA, reverse transcription of RNA viruses must always be performed. However, future technological advancements could make PCR redundant (via minimisation of starting DNA levels) or make future sequencing machines error-proof. Therefore, the model was used to examine the impact of such developments. Figure 7 shows that when the sample preparation PCR is removed, the peak of the mutation spectrum shifts substantially to lower frequencies. However, the high frequency end of the spectrum is still visible demonstrating that the RT process alone can generate observable high frequency mutations due to the skewed nature of its error distribution. When Illumina error is removed as well, the mutation spectrum changes drastically, with most genome positions (58%) having no observable error in the NGS reads. However, high frequency mutations up to the 0.5% threshold are again still observable again due to highly skewed error distribution of the RT process. Overall, this suggests that although PCR and Illumina machine error do contribute substantially to the mutation spectrum, removing them does not substantially affect the high frequency tail end of the spectrum or the minimum frequency thresholds. However, an error-proof sequencer capable of sequencing tiny amounts of DNA without PCR amplification would obviously be a major technological advancement. Although some sites would still have “high” frequency variants (due to the RT process), the majority of the genome will have no observable error, whilst only 5% of sites would have an observable error above 0.07%. This demonstrates that it is important to consider the overall error distribution of processes such as RT and PCR when selecting an appropriate frequency threshold and deciding upon the amount of error one is willing to tolerate. We investigated the effect of coverage and quality scores on the frequency threshold (Additional file 1), and found the 0.5% threshold to be stable at coverage’s of 10,000 and above, but the threshold does increase below this coverage. In addition, lowering the quality of the reads increases the threshold across all coverage levels, again highlighting that a threshold should be considered in context with all available information taken into account.

**Figure 8:**
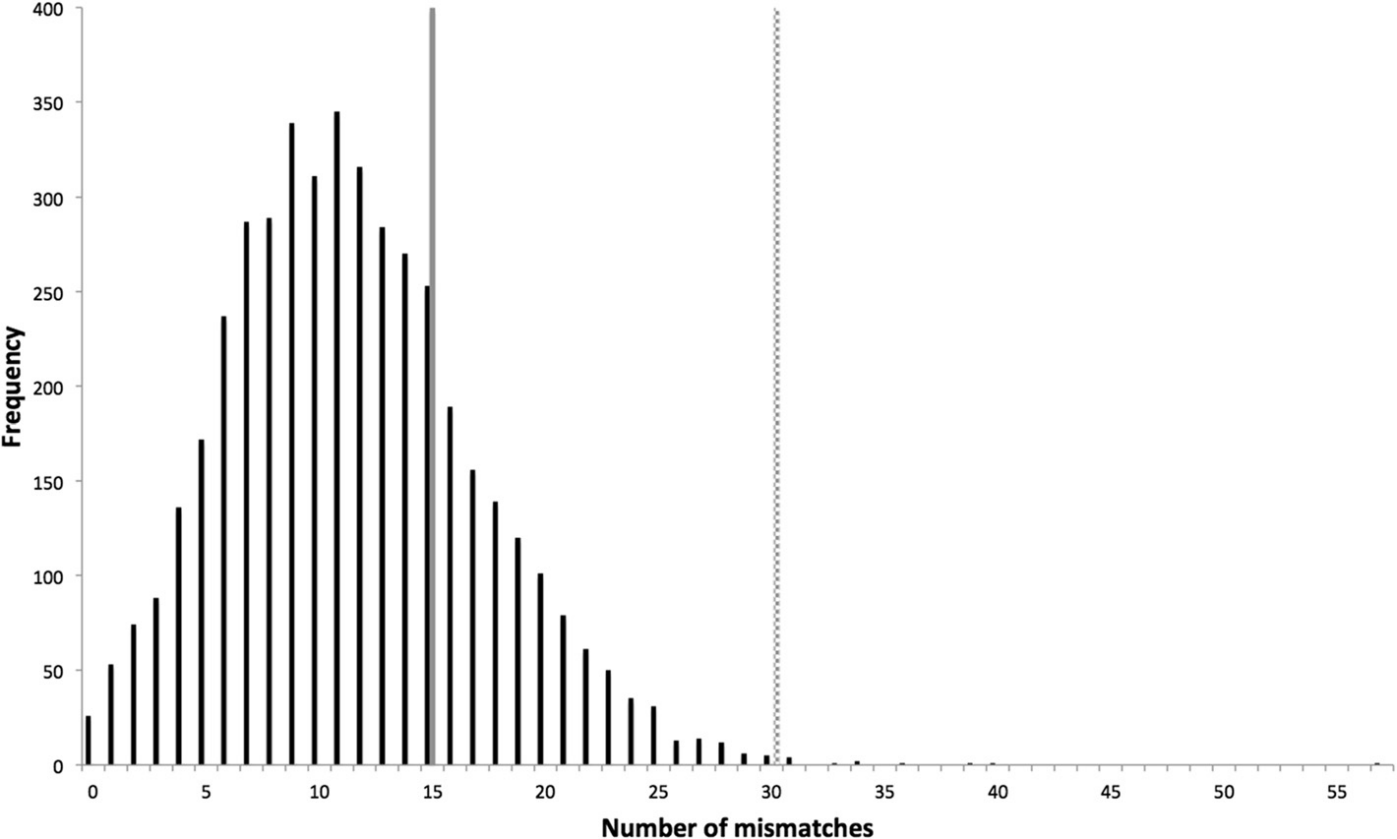
**Simulated number of mismatches from sequence and process error alone at a specified site.** For a given site, the model simulated 4,500 replicates, with each replicate having the same coverage (25,000) and average sequence error (0.000223) of the site, but receiving a random draw from the enzyme error distributions. The simulated number of mismatches for all replicates is presented as a histogram (black bars) that can then be compared to the real number of mismatches observed at the specified site. If one had observed 15 mismatches (grey bar) in the real biological data set, it is unlikely that any of these are real given the simulated distribution. However, if one observed 30 mismatches (grey dotted bar) it is much more likely that there is real biological variation at this site.

A secondary use of the model is to investigate whether the number of observed mutations at a given genome position of interest in a real viral data set is greater than would be expected from processing errors alone. The model is first provided with the genome position’s coverage and quality scores, along with the biological sample processing information. The model then utilises the previously determined RT and PCR enzyme error distributions to simulate the number of mutations that would be observed from processing errors alone at this position. The simulation is essentially replicated 4,500 times as each position in the model’s genome is set to have the given coverage and quality scores, but each position receives a random draw from the RT/PCR error distributions. The resulting histogram then displays the range of error mismatches that could be observed at this position given the variations due to sampling bottlenecks, stochasticity, and the enzyme error rates (Figure 8). This histogram can then simply be compared to observed number of mutations to determine how likely it is that they are real.

Figure 8 is an example of setting a frequency threshold in the context of a genome position of interest. In such a case, one already knows the coverage and quality scores, both of which can greatly affect a frequency threshold. Coverage directly affects frequency (at a coverage of 100 a single erroneous mutation would result in a frequency of 1% but is unlikely to be trusted), whilst a site with poor quality could similarly lead to a high error frequency from machine miscalls alone. Therefore, the model can be used to investigate the possible affect the RT and PCR processes are having at a specific site given what is already know from the sequence data. We envisage that the model would be used in this case for a select number of genome positions, such as though at the higher scale of the mutation spectrum, or specific sites of interest such as those that convey drug resistance, pathogenicity or become fixed later on in a transmission chain, due to the computational cost of running the model across the whole genome.

## Discussion

NGS technology provides the means to go beyond consensus sequences and investigate the population structure of viral samples. The introduction of erroneous mutations during the sample preparation process and miscalls during sequencing confounds the identification of true low frequency viral variants. Identifying such variants is important when tracing the source and development of specific mutations of interest, or when comparing the total diversity within viral populations, such as those before and after a bottleneck. In this study, we generated and analysed a collection of novel NGS data sets that reveal the amount of error introduced during each viral sample processing step and used them to set a minimum frequency threshold of ~0.5% to separate real mutations from process error. However, this threshold could be lowered at the cost of specificity, and will naturally vary depending on the specifics of the sample processing steps used such as enzyme fidelity and number of PCR cycles. We complemented this with a genome-scale model to gain a deeper understanding of each processing step.

The data and model show that despite high quality read filtering, Illumina error is still observable, although typically at relatively low frequencies. The introduction of PCR resulted in a substantial increase in the observable error to higher mismatch frequencies. However, using the highest fidelity DNApol enzymes and minimising the number of PCR cycles will logically reduce the amount of PCR error introduced. The model predicted the mean error rate of the DNApol used (Platinum Taq High Fidelity; Invitrogen) to be 2.64 × 10^−5^ substitutions per nt copied, which is only slightly higher than the manufacturers published error rate of 1.8 × 10^−5^ (6 times the error rate of standard taq; Invitrogen); a further study using novel BEAMing technology estimated a lower bound error rate of 1.4 × 10^−5^ [49]. However, the measured error rate is extremely dependent on the assay used, with estimates for Taq itself range from 1.1 × 10^−4^ [50] to between 2.3 × 10^−5^ [49] and 2.7 × 10^−5^ [51]. In this report, we have used a novel approach using NGS reads to estimate the error rate, and propose that the error rate is in fact best represented with a distribution rather than a single value. The model predicted a mean error rate of the RT enzyme used (Superscript III; Invitrogen) to be 6.13 × 10^−5^ substitutions per nt copied, which is within the range of published error rates for Superscript III (6.66 × 10^−5^ to 3.12 × 10^−5^, Invitrogen and [48]) which again varies depending on the assay used. Our RT-PCR sample was first transcribed into RNA using T7 polymerase (Ambion) before reverse transcription back into DNA. Therefore, the RT error rate estimated by the model is applied to both the transcription and reverse transcription steps as these two steps cannot be fitted separately with the available data. If the transcription step is less error prone, then the RT step will in fact have a higher error rate than we have reported here to compensate, and vice versa.

The model predicts that not all genome positions are equal and that some sites are much more prone to PCR error than others. This novel model prediction could be due to the sequence composition of surrounding bases influencing the likelihood of DNApol making an error, which has previously been reported for human polymerase *v* [52]. The model also makes a similar prediction for the RT process, which resulted in a highly skewed Beta distribution with some genome positions highly susceptible to error. Interestingly, this does not appear to be specific to the RT-PCR enzymes (Platinum Taq High Fidelity and Superscript III) or viral sequence (FMDV) that we used in this study. Similar findings have been reported with Hepatitis C virus [53], using different RT-PCR enzymes combined with a consensus sequencing strategy of numerous molecular clones. They reported that RT-PCR errors were not evenly distributed, but were concentrated in specific hotspot regions, one of which coincided with a known region of hyper-variability in the viral genome. This suggests that any specific sequence context or secondary structure that negatively impacts on the RT-PCR enzymes may also affect the viral RNA polymerase itself. Therefore, hyper-variable regions in viral genomes may be, in part, the result of natural polymerase mutational hotspots. Overall, identification of the sequence and structural signatures of mutational hotspot regions, and a comparison of these between different viruses would make an interesting study and could lead to novel insights into what drives the mutational dynamics of viruses.

From the data itself we identified a minimum frequency of ~0.5% above which one can be reasonably confident that an observed mutation is real. However, a minimum mismatch frequency threshold should be interpreted with some caution and must always be considered in context, as any mismatch frequency is highly dependent on the coverage at a genome position. For example, 1 observed mismatch at a genome position with a coverage of 100 leads to a mismatch frequency of 1% which would appear relatively high. However, a single mutation is not reliable given the error prone nature of the processes, and a coverage of 100 would be considered low compared to the average of 34,000 observed in our Seq control data set, suggesting an underlying problem with that genome position that has resulted in limited coverage. Additional filters should therefore be applied when validating the mismatches at a particular genome position, such as minimum coverage cut-offs, and validation against the number of mismatches expected given the sample processing procedure.

To address this, our model can also be used to investigate if the mutations observed at a given genome position are real, by simulating the number of mutations that would be expected from sequencing and sample processing error alone, and comparing this distribution to the observed number. This could be applied to any viral Illumnia data set, and adapted to represent alternative RT-PCR enzymes if estimates of their error rates are known; alternatively, one could simply run the model multiple times scanning through a range of enzyme error rates. Although we used 39 cycles of PCR in our optimised protocol, alternate virus/system protocols may well utilise fewer cycles. As shown in Figure 4, when the number of PCR cycles is halved, the mutation spectrum shifts substantially to lower error frequencies. Therefore, one should logically limit the number of PCR cycles used if possible as PCR errors accumulate with each cycle. Alternatively, there are high-fidelity polymerases available that have published error rates orders of magnitude above the Platinum Taq polymerase used in our protocol, which will also greatly reduce the observed errors. However, in both cases the RT step remains which alone can still result in erroneous high frequency variants, although only at relatively few genome positions. In addition, as Illumina machines share the same sequence by synthesis chemistry, and as we utilise the quality scores outputted by the sequencer itself, our results are applicable to more recent machines.

Recent techniques have been developed [54,55] that utilise the fragmentation followed by circularisation of viral RNA. Each small RNA circle is then reverse transcribed multiple times to create a single cDNA strand with multiple copies of the original RNA sequence. The cDNA can then be directly sequenced (after library adapter ligation) and as each read will contain multiple copies (typically three) of the same viral RNA sequence, reverse transcriptase errors and sequencer base miscalls can be readily identified; a similar strategy to that of utilising overlapping read pairs [9]. These approaches are very promising, however, they are currently limited to in-vitro samples due to the substantially large amount of initial viral RNA required, as they do not utilise PCR at any step. Therefore, our strategy still provides a valuable means for identifying and quantifying errors introduced during the processing of biological samples, such as field isolated during an epidemic, which typically have to amplified to achieve the required amount of RNA as standard. Furthermore, our results that reverse transcriptase is highly prone to error at some sites, may have implications for such circular re-sequencing techniques. An alternative approach to error handling is the incorporation of unique barcodes into sample DNA which have been successfully applied to viral population and fitness analyses [56,57].

Our work here has been focussed on the Illumina platform, but application of the same experimental techniques would be useful to assess and compare the error profiles, in terms of viral populations, of different platforms. It would also be possible to apply the models to other platforms, although this would likely involve substantial more work. Although a large proportion of the models, such as the RT-PCR component, could be directly applied to other sequencing platforms, the models would then need to be extended to represent each platform’s specific sample preparation protocol and sequencing error profile.

## Conclusions

We have created a novel set of laboratory control samples that enabled the level of error introduced by the RT and PCR processes to be assessed and minimum frequency thresholds to be set for true viral variant identification. We combined this with a genome-scale computational model of the sample processing and NGS calling process to gain a detailed understanding of the errors at each step, which predicted that RT and PCR errors are more likely to occur at some genomic sites than others. The model can also be used to investigate whether the number of observed mutations at a given site of interest is greater than would be expected from processing errors alone in any NGS data set. These data sets and models provide an effective means of separating true viral mutations from those erroneously introduced during sample processing and sequencing. Furthermore, the data sets themselves provide an ideal test set for the evaluation of viral variant calling tools to assess their ability to distinguish real viral variants from RT-PCR and sequencer errors.

### Availability of supporting data

The raw FASTQ files from all four control samples will be deposited and made publicly available from the Sequence Read Archive (SRA) at the European Nucleotide Archive (ENA).

## Additional files

Additional file 1:
**Additional description of the model fitting method and parameter estimation.**
