# Supplementary material for: Distinguishing low frequency mutations from RT-PCR and sequence errors in viral deep sequencing data
Source: BMC Genomics. 2015 Mar 24;16(1):229. doi: 10.1186/s12864-015-1456-x (PMC4425905; doi:10.1186/s12864-015-1456-x)
Supplement: Additional file 1: — Additional description of the model fitting method and parameter estimation. [file 12864_2015_1456_MOESM1_ESM.pdf]

Supplementary Information File 1

1: Alignment View

The genome-wide model operates at the population level. Instead of modelling 10^9 or 10^6 individual genomes (viral or plasmid) through the exponential PCR process, which would be computationally intensive, we view the population as an array or alignment of 10^6 initial genomes and operate on the numbers of As, Cs, Gs, and Ts present at each genome position. This population view is based on the assumption that the copying of each individual base in each genome during PCR (or RT) is independent and is therefore a single binomial trial with a probability of success (a mutation) equal to the DNA polymerase error rate. Therefore, with 10^6 genomes being duplicated in the first PCR cycle, each individual genome position is independently copied 10^6 times with a mutation potentially introduced each time.

We present an example of the alignment view of a viral population in Table S1 below. With a starting viral population of 10^6 genomes and with no viral diversity present, one would observe an alignment as in Step 1 where the reference base at each genome position is populated with 10^6 bases, whilst the remaining 3 non-reference bases each have a population of 0. A single cycle of the PCR process will duplicate the population at each genome position (Step 2). However, during the PCR copying process mutation errors could have been introduced into the population at each genome position. For example, assuming a DNA polymerase error rate of 1.1 x 10^{-5} mutations per nucleotide copied, one would expect 11 errors (10^6 x 1.1 x 10^{-5}) to be introduced at each genome position on average. However, the process is stochastic and therefore one may acquire more (or fewer) mutation errors at one position (Step 3). The extension of this process to more PCR cycles is then simple, with the viral population alignment undergoing duplication events with errors being introduction during the copying process determined by drawing from a binomial distribution.

**Step 1 – Initial State**					
**Position**	**1**	**2**	**3**	**…**	**N**
**Reference**	**T**	**A**	**G**	**…**	**C**
**A**	0	1000000	0	…	0
**C**	0	0	0	…	1000000
**G**	0	0	1000000	…	0
**T**	1000000	0	0	…	0
**Total**	1000000	1000000	1000000	…	1000000
**Frequency**	0	0	0	…	0
**Step 2 - Duplication**					
**Position**	**1**	**2**	**3**	**…**	**N**
**Reference**	**T**	**A**	**G**	**…**	**C**
**A**	0	2000000	0	…	0
**C**	0	0	0	…	2000000

**G**	0	0	2000000	…	0
**T**	2000000	0	0	…	0
**Total**	2000000	2000000	2000000	…	2000000
**Frequency**	0	0	0	…	0
**Step 3 – Error introduction**					
**Position**	**1**	**2**	**3**	**…**	**N**
**Reference**	**T**	**A**	**G**	**…**	**C**
**A**	2	1999987	8	…	1
**C**	7	0	0	…	1999989
**G**	2	13	1999991	…	3
**T**	1999989	0	1	…	7
**Total**	2000000	2000000	2000000	…	2000000
**Frequency**	5.50E-06	6.50E-06	4.50E-06	…	5.50E-06

Table S1: Model alignment view of PCR amplification with error. Step 1 represents the alignment of an initial starting viral population with no sample diversity present. Step2 represents a PCR duplication of the population in Step 1 with no error introduced. Step 3 represents a PCR duplication of Step 1 with errors introduced, with the number of errors made at each genome positions determined by drawing from a binomial distribution.

2: PCR Efficiency

Although the PCRhigh sample underwent 39 cycles of PCR, the PCR fragments generated are long (over 4,000bp) and our PCR efficiency experiment show that PCR efficiency is at 80% during the initial stages (Figure S1). The efficiency of the PCR process deteriorates with each cycle, and this process can be effectively modelled with a classical PCR sigmoid function [1] when combined with knowledge of starting genome copy number and maximum genome copy number.

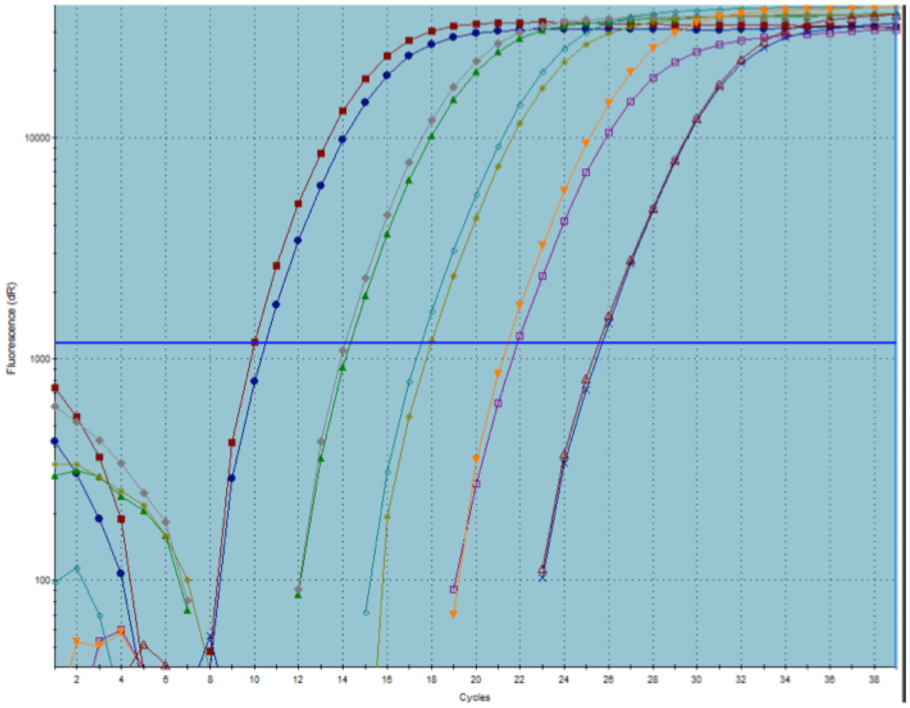

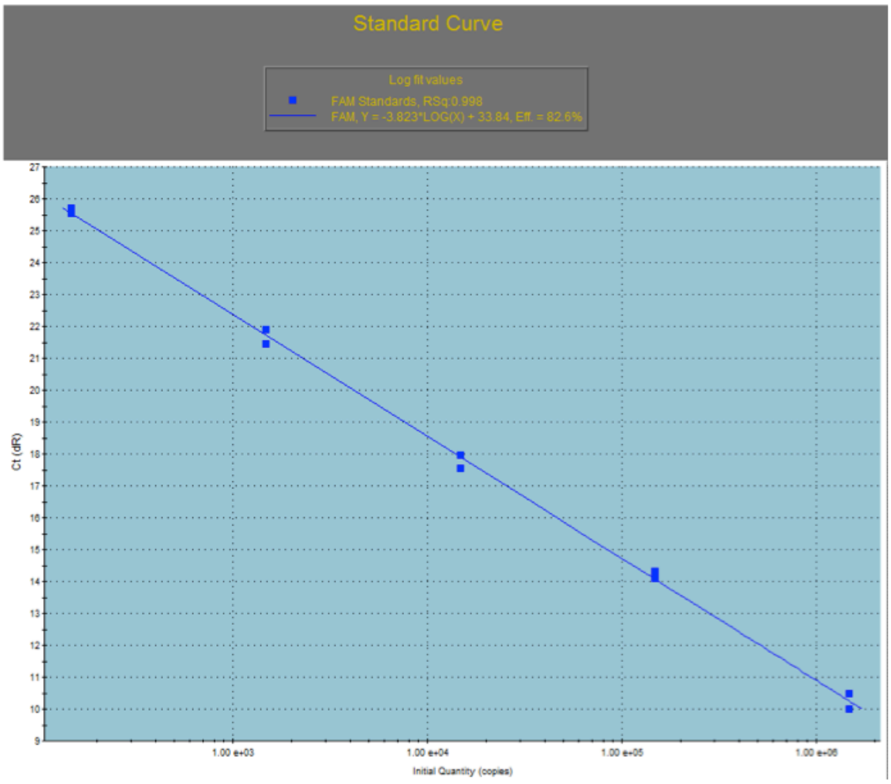

Figure S1. PCR efficiency calculation: (Top) A 5-point standard curve for the amplification of PCR fragment one (PCR1), using a log serial dilution series of pT7S3 plasmid in carrier RNA from an initial concentration of 1.47×10^6. All reactions were performed in duplicate using 1µl of High Fidelity Platinum Taq DNA polymerase (Invitrogen), EvaGreen fluorescent dye (Biotum) at a concentration of 0.125mM, dNTPS at a concentration of 0.2mM and MgCL2 at a concentration of 2mM. Primers O1BFS 516+F and O1BFS 4563R, had a final concentration of 0.2uM. All reactions were run on an MX3005 (Agilent) using the following cycling conditions, 95 °C initial

denaturation, followed by 39 cycles of; Denaturation at 94 °C for 30 seconds, Annealing at 55 °C for 30 seconds and Extension at 72 °C for 4 minutes, this was followed by a final extension at 72 °C for 8 minutes. (Bottom) C_t values were plotted against log copy numbers to calculate the efficiency of amplification for each fragment using the MxPro software (v4.1.build 389, Stratagene). The percentage amplification efficiency for this PCR was 82.6%. All negatives controls were negative.

We know that our starting copy number was 10^6 plasmid DNA genomes. We can calculate our maximum copy number by calculating the maximum number of genomes that could possibly be created from the available deoxyribonucleotides (dNTPs) present in the PCR reaction tube. The 50 µl PCR master mix contained 1µl of 10mM dNTPs, which equates to 6.02 x 10^{15} molecules of each dNTP (using Avogadro's number of 6.02 x 10^{21}). Therefore, a maximum of 6.02 x 10^{12} PCR fragments could be created (6.02 x 10^{15} / [4000bp /4]). This equates to a maximum of 22 effective PCR duplication cycles (10^6 x 2^{22}), therefore it would only have been possible for the starting population to be duplicated a maximum of 22 times. However, depletion of dNTPs will have begun before then severely hindering the completion of full PCR products. To take this into account, we therefore represent the PCR process as going through 21 effective duplication cycles (from the original 39 laboratory cycles) for the PCRhigh model resulting in 10^6 x 2^{21} = 2.1 x 10^{12} full PCR products.

Rather than using the above calculation of 21 effective PCR cycles, one can alternatively utilise classical PCR efficiency formulas based on fluorescence [1]:

(1) $F_c = \frac{F_{\max}}{1 + e^{-(C - C_{1/2}/k)}} + F_b$

Where C is cycle number, F_c is the reaction fluorescence at cycle C, F_{max} is the maximum reaction fluorescence that defines the cessation of amplification, $C_{1/2}$ is the fractional cycle at which reaction fluorescence reaches half of F_{max}, k is the slope of the curve and F_b is the background fluorescence. When C=0, F_0 can be derived from the equation 1 as:

(2) $F_0 = \frac{F_{\max}}{1 + e^{(C_{1/2}/k)}}$

Rather than fluorescence values, one can adapt these formulae to consider the number of genomes. Where C is cycle number, F_c is the number of genomes at cycle C, F_{max} is the maximum number of genomes that defines the cessation of amplification, $C_{1/2}$ is the fractional cycle at which the number of genomes is half of F_{max}, k is the slope of the curve, F_0 is the starting number of genomes and F_b is equivalent the number of background genomes which is 0.

We already know F_0 (10^6) and F_{max} (2.1 x 10^{12}) but we do not know k or $C_{1/2}$. However, rearranging equation 2 gives us:

(3) $k = \frac{C_{1/2}}{\ln(\frac{F_{max}}{F_0} - 1)}$

Therefore, if we know $C_{1/2}$ we can calculate k. We can estimate $C_{1/2}$, and therefore k, by using the equations above to simulate different PCR amplification and therefore efficiency curves, with a target of achieving a PCR efficiency at cycle 1 of ~80% to match our laboratory data. We find that with the defined F_0 and F_{max} values, a $C_{1/2}$ of 25, and therefore a k of 1.717, results in an initial efficiency of approximately 80% (Figure S2).

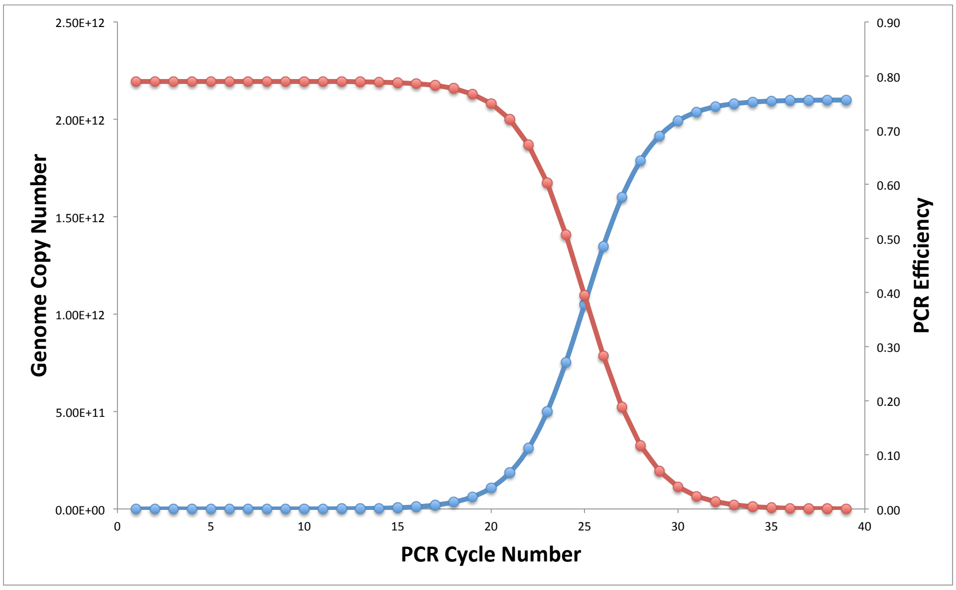

Figure S2: Simulated PCR amplification and efficiency curves. The blue line is the PCR amplification curve and represents the number of genomes (genome copy number, left y-axis) present after each PCR cycle. The red line is the PCR efficiency curve and represents the efficiency of the PCR process at each cycle (right y-axis); the efficiency at cycle c is calculated as $E_c = (F_c/F_{c-1})-1$, where F_c and F_{c-1} are the genome copy numbers at cycle c and c-1 respectively.

PCR efficiency can then become part of the simulation model, with the efficiency at each cycle determining the number of bases at each genome position that are duplicated this cycle (see Methods section of main paper). Alternatively, one can simply utilise the number of effective cycles of duplication that occur during the PCR process after taking efficiency into account, for PCRhigh there are 21 effective cycles of duplication.

3: Illumina Library Prep PCR

There are 10 cycles of PCR during the Illumina library preparation using the DNA polymerase Phusion Oy (Finnzymes) which has a published error rate of 4.4×10^{-7} (1 in 2,272,727). This error rate is not fitted by the model as we do not have a dataset without the Illumina Library PCR and are therefore unable to fit parameters effectively for this step. However, this step is predicted to have a minor impact on the process induced error during to the high fidelity of the enzyme.

For example, with a genome position with a starting population (P_0) of 10^6, an enzyme error rate (E_{rr}) of 4.4×10^{-7}, after 10 (c) PCR cycles would have approximately 2,253 errors (a frequency of 2.2×10^{-6}):

Population size after 10 cycles
$P_{10} = P_0 \times 2^c = 10^{6 \times} 2^{10} = 1.024 \times 10^9$

Correct bases after 10 cycles
$R_{10} = P_0 \times (2 - E_{rr})^c = 10^6 \times (2 - 4.4\text{x}10^{-7})^{10} = 1{,}023{,}997{,}747$

Incorrect bases after 10 cycles
$I_{10} = P_{10} - R_{10} = 1.024 \times 10^9 - 1{,}023{,}997{,}747 = 2{,}253$

The average coverage of our NGS data sets was 34,000. We can use a hypergeometric distribution to calculate the probability of observing Illumina PCR errors in the coverage of 34,000 via 34,000 random draws from a population with 2,253 "white" balls (error bases) and 1,023,997,747 black balls (correct bases). In such a case, the probability of observing 0 errors is 0.9279, the probability of observing 1 error is 0.0694, and the probability of observing 2 errors is 0.0026. Therefore, the vast majority (93%) of genome positions will have no observable error as a result of the Illumina library preparation PCR. Whilst under 7% of sites will have 1 observed error, and less than 0.3% of sites will have 2 observed errors.

4. Genome-wide model fitting

For the genome-wide model fitting and parameter estimation, we use a Sequential Monte Carlo (SMC) algorithm [2-4], a form of Approximate Bayesian Computation (ABC). In SMC, a number of parameters $\{\theta^1,\ldots,\theta^N\}$ which are called particles are sampled from the prior distribution $\pi(\theta)$ and propagated through a sequence of intermediate distributions $\pi(\theta|d(x_0,x^*)\leq\varepsilon_i)$ until it represents a sample from the target distribution $\pi(\theta|d(x_0,x^*)\leq\varepsilon_T)$. The tolerances ε_i are chosen such that $\varepsilon_i > \ldots > \varepsilon_T \geq 0$, thus the distributions gradually evolve towards the target posterior. A standard SMC algorithm proceeds as follows, taken from [3]:

1) Initialise $\varepsilon_1,\ldots,\varepsilon_T$ and set the population indicator t=0
2) Set the particle indicator i=1 with a target of selecting N particle
3) If t=0, sample θ^{**} independently from $\pi(\theta)$. Else, sample θ^* from the previous population $\left\{\theta_{t-1}^{(i)}\right\}$ with weights w_{t-1} and perturb the particle to obtain $\theta^{**} \sim K_t(\theta|\theta^*)$, where K_t is a perturbation kernel.

4) If $\pi(\theta^{**})$=0 return to (3) else simulate a candidate dataset $x^* \sim f(x|\theta^{**})$. If $d(x^*, x_o) \geq \varepsilon_t$ return to (3)
5) Set $\theta_t^{(i)} = \theta^{**}$ and calculate the weight for particle $\theta_t^{(i)}$. If t=0, $w_t^{(i)} = 1$ else

$$w_t^{(i)} = \frac{\pi\left(\theta_t^{(i)}\right)}{\sum_{j=1}^{N} w_{t-1}^{(j)} K_t\left(\theta_{t-1}^{(j)}, \theta_t^{(i)}\right)}$$

6) If i<N set $i=i+1$ and go to (3)
7) Normalize weights
8) If t<T set $t=t+1$ and go to (2)

Particles sampled from the previous distribution are denoted by a single asterisk, and after perturbation these particles are denoted by a double asterisk. In the genome-wide model, uniform distributions are used for all priors, and a Gaussian distribution is used for the perturbation kernel K_t, with a variance twice that of the empirical variance of the simulated points [4]. We manually initialise the tolerance ε_1 at the start and then dynamically set subsequent tolerances to be the median distance $d(x^*, x_o)$ of the previous population. The SMC process essentially fits the model to the given data set and thus provides estimates of the error rates of the enzymes used during RT and PCR, as well as the underlying distributions involved.

5. Simulation Model Runs

For the PCRhigh model, only two parameters are fit by the model – the alpha and beta parameters of the beta distribution that represents the variation in the DNA polymerase error rate at different genome positions. Figure S3 represents a 2D density contour plot of the relationship between these two parameters from all the particles present in the final SMC population. In this population, the average values for the alpha and beta parameters were 2.01 and 76,254 respectively.

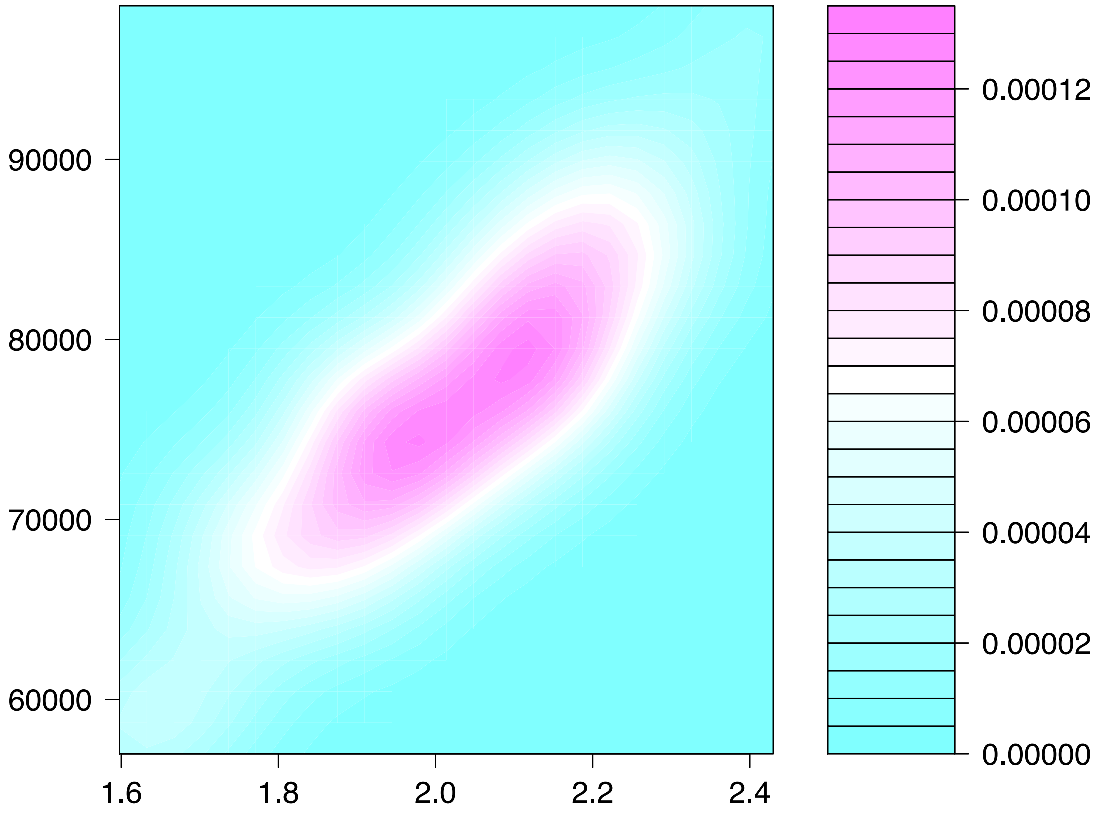

Figure S3: PCRhigh model beta distribution parameters for DNA polymerase error. The x and y axes represent the alpha and beta parameters of the beta distribution representing the error rate of the DNA polymerase, respectively. Only parameter particles from the final target population of the SMC run were used. The plot was generated using the kde2d (two-dimensional kernel density estimation) and contour functions in R.

For the RT-PCR model, only two parameters are fit by the model – the alpha and beta parameters of the beta distribution that represents the variation in the reverse transcriptase error rate at different genome positions. Figure S4 represents a 2D density contour plot of the relationship between these two parameters from all the particles present in the final SMC population. In this population, the average values for the alpha and beta parameters were 0.19 and 3,074 respectively.

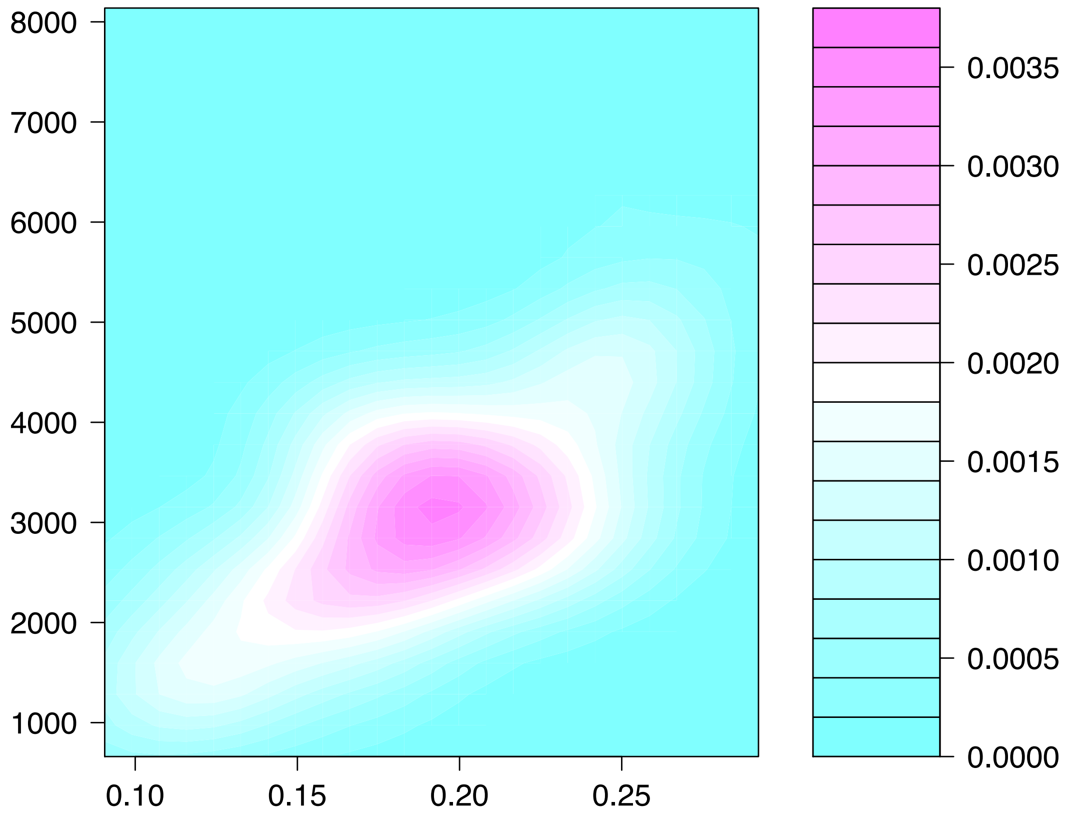

Figure S4: RT-PCR model beta distribution parameters for reverse transcriptase error. The x and y axes represent the alpha and beta parameters of the beta distribution representing the error rate of reverse transcriptase, respectively. Only parameter particles from the final target population of the SMC run were used. The plot was generated using the kde2d (two-dimensional kernel density estimation) and contour functions in R.

6: Mutation classification

All mismatches to the reference sequence present in the analysed clonal data sets were characterised (Figure S5). The Seq control data set, whose only source of error is from the Illumina machine, has 40% of all base miscalls occurring at G positions with a G to T miscall observed most often. In the PCRhigh and RT-PCR data sets, transitions are observed far more frequently than transversions, as expected. However, the most favoured transversion is the G to T suggesting the base miscalls from the Illumina machine are still a factor in these data sets.

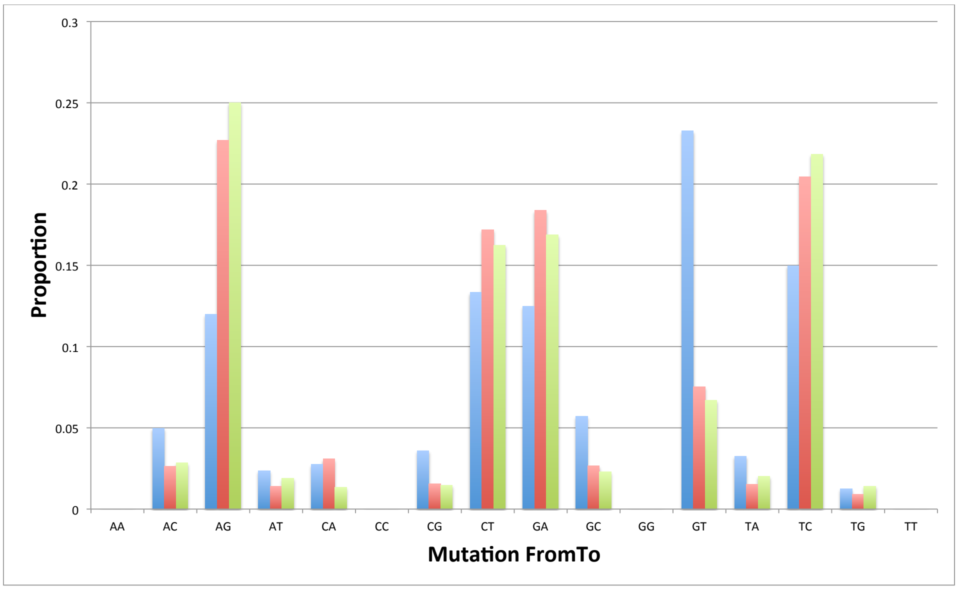

Figure S5: Classification of mutations: All the mismatches to reference sequence present in the Seq control (blue), PCRhigh (red) and RT-PCR (green) data sets were classified based on the From (reference base) and To (mismatched read base) bases of the mismatch.

7: GC influence on coverage

The effect of GC content on read coverage across the genome was investigated (Figure S6). Variations in coverage across the genome appears to correlate with variations in GC content, with sharp changes in coverage occurring when there are sharp changes in GC content. GC content is thought to influence coverage via amplification bias during the Illumina library preparation PCR and possibly sonication bias [5].

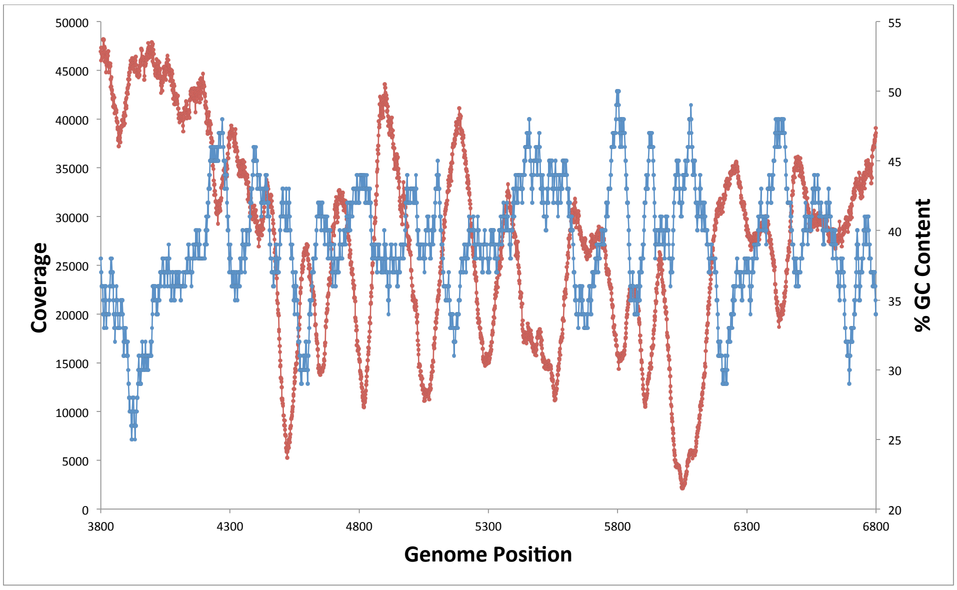

Figure S6: GC content vs coverage. GC content (blue) was calculated as the percentage of G and C bases in a sliding window of 73bp (a read length) across the genome and plotted along with read coverage (read) across the genome.

7: Thresholds

For each sample, genome positions were sorted in ascending order based on their mismatch frequency to create a mismatch profile to investigate the setting of appropriate error thresholds. No genomes positions have a mismatch frequency above 0.657% in the RT-PCR sample (Figure S7 below; Table 1 main text). Therefore, all variants in the real FMDV sample observed above this threshold are highly likely to be true. However, this threshold comes with a cost in that a number of true variants in the real FMDV sample will be classed as errors. Conversely, lowering the threshold to the 0.14% (the 95th percentile of the RT-PCR sample) will identify more variants in the real FMDV sample, but at a cost of identifying 5% of the errors incorrectly as true variants.

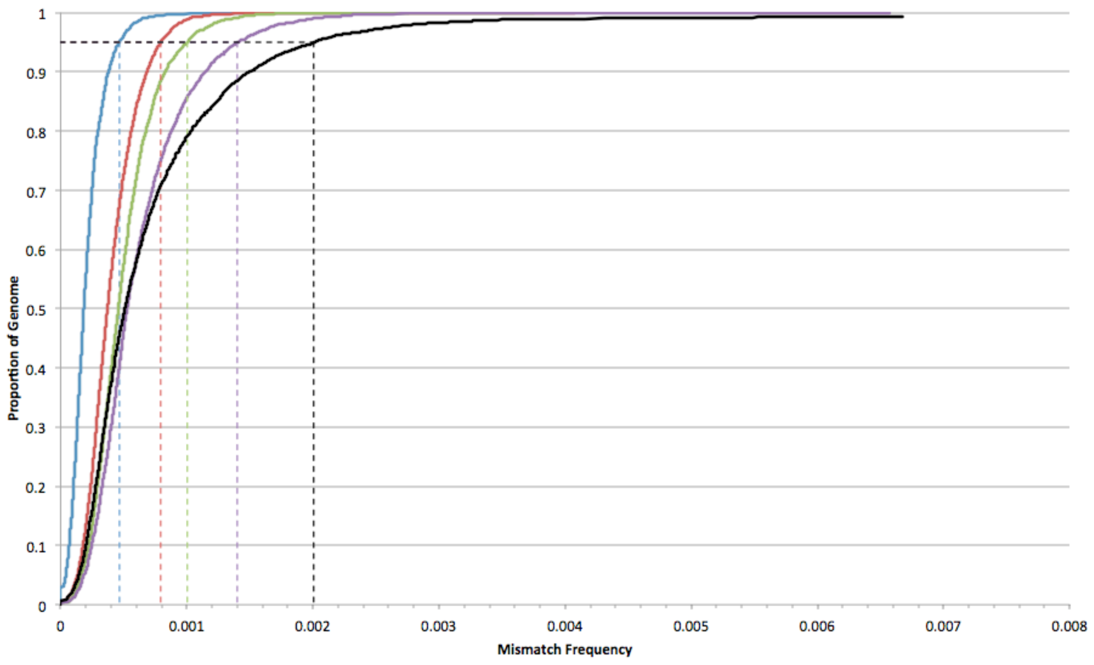

Figure S7: Mismatch frequency profile. For each sample, genome positions were sorted in ascending order based on their mismatch frequency, and the proportion of the genome positions (y-axis) is then plotted against mismatch frequency (x-axis) to create the profile for the sequence (blue), pcr-low (red), pcr-high (green) and rt-pcr (purple) control samples as the real FMDV sample (black). Dashed lines represent the frequency threshold at the 95th percentile for each sample.

Figure 8 thresholds

8: LoFreq Comparison

For a comparison to existing variant callers, we ran our RT-PCR control sample through the variant calling program lofreq (http://csb5.github.io/lofreq/). Lofreq is a fast and sensitive variant calling program that makes full use of base-call qualities and other sources of errors inherent in sequencing (e.g. mapping or base/indel alignment uncertainty), which are usually ignored by other methods or only used for filtering. However, lofreq does not consider RT-PCR errors. Our RT-PCR control panel stems from a DNA plasmid (containing a full-length FMDV genome) grown in bacteria, before undergoing transcription, reverse transcription, and then 39 cycles of PCR amplification before undergoing ultra-deep sequencing. Given the error rate of bacterial DNA replication, practically all mutations observed in the reads for the RT-PCR control sample will be errors generating during sample preparation of machine sequencing errors, and not real mutations. However, lofreq identifies a total of 537 variants in the RT-PCR control sample, within the specific genome region considered. These variants range from 0.06% to 0.6% with a median of 0.1106% and a mean of 0.1177%. Overall, this demonstrates the importance of considering RT-PCR errors when variant calling, otherwise erroneous variants could well be called as true. However, it is important to note that the amount of error introduced by the RT-PCR process will

vary depending on the fidelity of the enzymes used as well as the number of PCR cycles. Furthermore, RT-PCR errors are unlikely affect variant calling at high frequency, such as those above 1%.

9: Coverage influence

We investigated the effect of coverage and quality scores on the frequency threshold (Figure S8), and found the ~0.5% threshold to be stable at coverage's of 10,000 and above, but the threshold does increase below this coverage. In addition, lowering the quality of the reads increases the threshold across all coverage levels, again highlighting that a threshold should be considered in context with all available information taken into account.

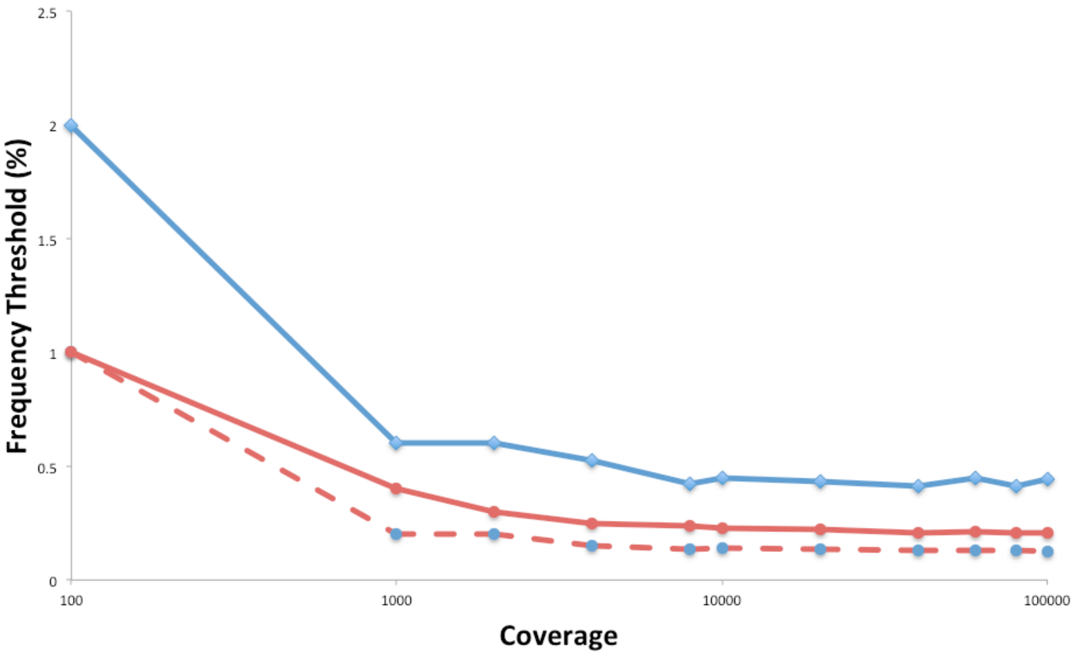

Figure S8: Coverage and quality effect on thresholds. The model was run at various coverage's (x-axis) to investigate it's effect on the observed mismatch frequency and therefore the threshold used to identify errors (y-axis). The solid blue and red lines represent the maximum and 95th percentile mismatch frequencies, respectively, and utilised an average probability of error (quality scores) of 0.00223. Whilst the dashed red line represent the 95th percentile mismatch frequency at various coverage values but with an average probability of error of 0.001 (Q30).

10: References

1. Rutledge RG (2004) Sigmoidal curve-fitting redefines quantitative real-time PCR with the prospective of developing automated high-throughput applications. Nucleic Acids Res 32: e178.
2. Sisson SA, Fan Y, Tanaka MM (2007) Sequential Monte Carlo without likelihoods. Proc Natl Acad Sci U S A 104: 1760-1765.

3. Toni T, Welch D, Strelkowa N, Ipsen A, Stumpf MP (2009) Approximate Bayesian computation scheme for parameter inference and model selection in dynamical systems. J R Soc Interface 6: 187-202.
4. Beaumont M, Cornuet JM, Marin JM, Robert CP (2009) Adaptive approximate Bayesian computation. Biometrika 96: 983-990.
5. Aird D, Ross MG, Chen WS, Danielsson M, Fennell T, et al. (2011) Analyzing and minimizing PCR amplification bias in Illumina sequencing libraries. Genome Biol 12: R18.
